# Effects of Vitamin D Supplementation on Serum 25-Hydroxyvitamin D Concentrations in Cirrhotic Patients: A Randomized Controlled Trial

**DOI:** 10.3390/nu8050278

**Published:** 2016-05-10

**Authors:** Stefan Pilz, Csilla Putz-Bankuti, Martin Gaksch, Walter Spindelboeck, Marius Haselberger, Florian Rainer, Andreas Posch, Philipp Kreuzer, Tatjana Stojakovic, Vanessa Stadlbauer, Barbara Obermayer-Pietsch, Rudolf E. Stauber

**Affiliations:** 1 Division of Endocrinology and Diabetology, Department of Internal Medicine, Medical University of Graz, Auenbruggerplatz 15, 8036 Graz, Austria; martin.gaksch@gmail.com (M.G.); barbara.obermayer@medunigraz.at (B.O.-P.); 2 Department of Internal Medicine, Landeskrankenhaus Hoergas-Enzenbach, Hörgas 30, 8112 Gratwein-Straßengel, Austria; Csilla.Putz-Bankuti@lkh-hoergas.at (C.P.-B.); philipp.kreuzer@klinikum-graz.at (P.K.); 3 Division of Gastroenterology and Hepatology, Department of Internal Medicine, Medical University of Graz, Auenbruggerplatz 15, 8036 Graz, Austria; walter.spindelboeck@medunigraz.at (W.S.); marius.haselberger@stud.medunigraz.at (M.H.); flo_rainer@hotmail.com (F.R.); andreas.posch@medunigraz.at (A.P.); vanessa.stadlbauer@medunigraz.at (V.S.); 4 Clinical Institute of Medical and Chemical Laboratory Diagnostics, Medical University of Graz, Auenbruggerplatz 15, 8036 Graz, Austria; tatjana.stojakovic@medunigraz.at

**Keywords:** vitamin D, RCT, supplementation, intervention, liver

## Abstract

*Background*: The liver is crucial for 25-hydroxyvitamin D (25(OH)D) metabolism, and vitamin D deficiency is highly prevalent in patients with cirrhosis and predicts adverse outcomes. We aimed to evaluate whether vitamin D supplementation in patients with cirrhosis is effective in increasing 25(OH)D serum concentrations. Secondary outcome measures included liver function tests (aspartate aminotransferase (AST), alanine aminotransferase (ALT), gamma glutamyltransferase (GGT), and alkaline phosphatase (AP)), albumin, International Normalized Ratio (INR), bilirubin, the liver fibrosis marker hyaluronic acid, and parameters of mineral metabolism including parathyroid hormone (PTH). *Methods*: This is a double-center, double-blind, placebo-controlled study conducted from December 2013 to May 2014 at the Medical University of Graz, and the hospital Hoergas-Enzenbach, Austria. We enrolled 36 consecutive patients with cirrhosis and 25(OH)D concentrations below 30 ng/mL. Study participants were randomly allocated to receive either 2800 International Units of vitamin D3 per day as oily drops (*n* = 18) or placebo (*n* = 18) for 8 weeks. *Results*: Thirty-three study participants (mean (SD) age: 60 (9) years; 21% females; 25(OH)D: 15.6 (7.4) ng/mL) completed the trial. The mean treatment effect (95% CI) for 25(OH)D was 15.2 (8.0 to 22.4) ng/mL (*p* < 0.001). There was no significant effect on any secondary outcome. *Conclusions*: In this randomized controlled trial, vitamin D supplementation increases 25(OH)D serum concentrations, even in cirrhotic patients.

## 1. Introduction

Vitamin D is important for calcium homeostasis and ensures adequate calcium supply for bone mineralisation by its effects on bone, kidney, and gut [1,2,3,4]. The liver plays a crucial role in vitamin D metabolism because hepatic 25-hydroxylation, catalyzed by cytochrome P450 2R1 (CYP2R1), is required to convert vitamin D to 25-hydroxyvitamin D (25(OH)D) [1,2]. 25(OH)D is the main circulating vitamin D metabolite that is used to classify vitamin D status and that best reflects vitamin D supply by nutrition, supplements, and UV-B-induced vitamin D synthesis in the skin [3,4]. Beyond the classic effects of vitamin D on bone and mineral metabolism, accumulating evidence shows that vitamin D deficiency is a risk factor for a variety of chronic diseases and mortality [3,4,5,6,7,8,9,10,11]. Whether vitamin D deficiency is simply the consequence of various extra-skeletal diseases and risk factors or may even contribute to adverse outcomes is, however, largely unclear [3,4,5,6,7,8,9,10,11].

Patients with liver diseases are at a particularly high risk of vitamin D deficiency and it has been documented that low 25(OH)D concentrations are associated with liver dysfunction and mortality [1,2,12,13,14,15]. Decreased 25(OH)D levels have also been observed in patients with non-alcoholic fatty liver disease (NAFLD), and several epidemiological and experimental studies suggest that vitamin D might be useful for the treatment of liver fibrosis [1,2,14,15,16]. Interventional studies on vitamin D supplementation in patients with liver diseases are sparse and have shown mixed results on the effects of vitamin D on parameters of mineral metabolism, liver function, and fibrosis [1,2,17,18,19,20,21,22,23,24].

To the best of our knowledge, there is no randomized placebo controlled trial published on vitamin D supplementation in a cohort of patients with cirrhosis. Such a randomized controlled trial (RCT) on the effects of vitamin D supplementation on 25(OH)D concentrations and parameters of liver function and fibrosis is, however, important to evaluate vitamin D effects in the setting of cirrhosis in order to guide clinical decisions and provide a basis for the development of vitamin D recommendations in these patients. We therefore performed a RCT in cirrhotic patients to evaluate the effects of vitamin D supplementation on 25(OH)D, parameters of liver function and synthesis, and hyaluronic acid as a marker of liver fibrosis [25]. 

## 2. Materials and Methods

### 2.1. Study Design

This study was sponsored by the Medical University of Graz, Austria, and was designed as a double-center, double-blind, placebo-controlled, parallel-group study conducted at the Medical University of Graz, Austria and at the hospital Hoergas-Enzenbach, Gratwein, Austria. The reporting of this trial follows the recommendations of the CONSORT (Consolidated Standards of Reporting Trials) 2010 statement [26]. This trial was registered at clinicaltrials.gov (ClinicalTrials.gov Identifier NCT02009748) [27]. 

### 2.2. Participants

We enrolled consecutive patients with compensated cirrhosis attending the liver clinic at one of the two study centers. Diagnosis of cirrhosis was based on clinical and radiological features or on liver histology. Further inclusion criteria were 25(OH)D below 30 ng/mL (multiply by 2.496 to convert ng/mL to nmol/L), age between 18 and 75 years, and a negative pregnancy test in women of childbearing potential. Exclusion criteria were the presence of hepatocellular carcinoma, hypercalcemia (plasma calcium concentrations above 2.65 mmol/L), pregnancy or lactating women, drug intake as part of another clinical study, estimated glomerular filtration rate (eGFR) according to the Modification of Diet in Renal Disease (MDRD) formula below 15 mL/min/1.73 m^2^, any clinically significant acute disease requiring drug treatment, regular intake (in addition to study medication) of more than 800 International Units (IU) of vitamin D daily during the last four weeks before study entry. All study participants gave written informed consent and the study was approved by the ethics committee at the Medical University of Graz, Austria. The study was designed to comply with the Declaration of Helsinki.

Recruitment of study participants and all study procedures were performed by the staff at our two study centres: the outpatient clinics of the Division of Gastroenterology and Hepatology, Department of Internal Medicine, Medical University of Graz, and of the hospital Hoergas-Enzenbach, Gratwein, Austria. Patients were informed about this study by either a conversation in the outpatient clinics or a telephone call. The study was conducted from December 2013 to May 2014.

### 2.3. Intervention

Study medication was filled into numbered bottles according to a computer-generated randomization list. Randomization was done by use of web based software with GCP compliance as confirmed by the Austrian Agency for Health and Food Safety (AGES) [28]. Eligible study participants were randomly allocated in a 1:1 ratio to 2800 IU vitamin D_3_ as seven oily drops per day (Oleovit D3, Fresenius Kabi Austria, 8055 Graz, Austria; 1 bottle contains 180,000 IU vitamin D3 in 12.5 mL) or matching placebo as seven oily drops per day for 8 weeks. The dose of 2800 IU vitamin D per day was chosen because a rule of thumb suggests that vitamin D supplementation of 1000 IU increases 25(OH)D levels by approximately 10 ng/mL. Given that a commonly-used normal range of 25(OH)D is 30 to 60 ng/mL, we concluded that a supplementation of 2800 IU daily may be sufficient to increase the 25(OH)D level of most study participants to target ranges without causing supraphysiological 25(OH)D levels. We performed a permuted block randomization with a block size of 10 and no further stratification. All investigators who enrolled participants, collected data and assigned intervention were blinded to the allocation of the study patients. 

### 2.4. Primary Outcome Measure

The primary outcome measure was the between-group difference in serum 25(OH)D. 

### 2.5. Secondary Outcome Measures

Secondary outcome measures included between-group differences in liver function tests (*i.e.*, aspartate aminotransferase (AST), alanine aminotransferase (ALT), gamma glutamyltransferase (GGT), and alkaline phosphatase (AP)), albumin, International Normalized Ratio (INR), bilirubin, and the liver fibrosis marker hyaluronic acid [25]. All of these outcome measures were pre-specified before study start.

In addition to these pre-specified outcomes, we tested for further effects of vitamin D supplementation on parameters of mineral metabolism, *i.e.*, parathyroid hormone (PTH), total plasma calcium, free plasma calcium, urinary midstream calcium to creatinine ratio (in mmol/L divided by mmol/L), and plasma phosphate. The decision to test for these parameters of mineral metabolism was made after the study was finished but before unblinding the data. All outcome parameters were assessed in the morning before treatment started and just after the 8 week treatment period.

### 2.6. Measurements

Blood samplings were collected in the morning after an overnight fast. Serum 25(OH)D was measured by means of a ChemiLuminescence assay (IDS-iSYS 25-hydroxyvitamin D assay; Immunodiagnostic Systems Ltd., Boldon, UK) on an IDS-iSYS multidiscipline automated analyser, with an intra- and inter-assay coefficient of variation (CV) of 6.2% and 11.6%, respectively. PTH was determined with a sandwich ElectroChemiLuminescence Immunoassay on an Elecsys 2010 (Roche Diagnostics, Mannheim, Germany) with an intra- and inter-assay CV of 1.6% and 3.9%, respectively. We calculated the estimated GFR according to the MDRD formula [29]. Serum levels of hyaluronic acid were measured as part of the Enhanced Liver Fibrosis (ELF) score using the ELF test by Siemens Healthcare Diagnostics Inc. on an ADVIA Centaur^®^ immunoassay system (Siemens Medical Solutions Diagnostics Inc., Tarrytown, NY, USA) [30]. All other parameters were determined by routine laboratory procedures.

Child-Pugh score and the model for end-stage liver disease (MELD) score were used to determine the severity of cirrhosis [31,32].

### 2.7. Statistical Analysis

Sample size calculation was based on previously published data on 25(OH)D serum levels in cirrhotic patients at our center [12]. The primary endpoint is the 25(OH)D level at study end. We assumed 25(OH)D levels of 15 ± 10 ng/mL at baseline and in the placebo group at study end and of 30 ± 20 ng/mL in the intervention group after vitamin D supplementation. For a two-sample analysis with an alpha of 5% and a power of 95%, we calculated a sample size of 26 study participants per group. To compensate for drop-outs during the study, we planned to enroll 30 patients per group. 

Normally distributed continuous variables are presented as means (with standard deviation, SD) and skewed variables as medians (with interquartile range). Categorical data are shown as percentages. Skewed variables were log(e) transformed before they were used in parametric statistical analyses. Comparisons between the placebo and intervention group at baseline were performed by unpaired student’s *t*-test and by Chi Square or Fisher’s exact test. Analyses of outcome measures were performed according to the intention-to-treat principle with no data imputation and inclusion of all study participants with baseline and follow-up values of the respective outcome variable. We used Analyses of Covariance (ANCOVA) with adjustments for baseline values to test for differences in the outcome variables between the intervention and the placebo group at the follow-up visit [33]. A *p*-value below 0.05 was considered statistically significant. Statistical analyses were performed by using SPSS version 22.0 software (SPSS, Chicago, IL, USA).

## 3. Results

**Figure 1:**
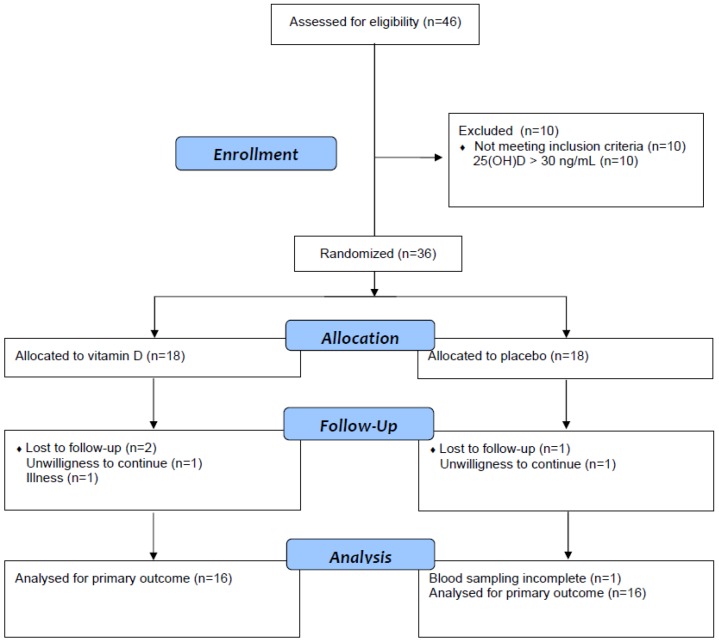
Participant flow chart.

A total of 46 patients gave written informed consent and were assessed for eligibility. The participant flow through the study is shown in Figure 1. Instead of 60 patients according to our statistical power calculation, we were only able to randomize 36 patients. We decided to stop the study enrollment after 36 patients (prior to unblinding) because we had no further funding to continue the study. The first patient was randomized in December 2013 and the last follow-up visit was performed in May 2014. 

Baseline characteristics of all randomized study participants are shown in Table 1. Cirrhosis was due to ethanol in 23 patients, nonalcoholic steatohepatitis in 7 patients, hepatitis C virus in 5 patients, and hemochromatosis in 1 patient. There was no significant difference between the vitamin D and the placebo group for all study characteristics in Table 1. Of the 36 randomized study participants, 26 (72%) had 25(OH)D levels below 20 ng/mL and 14 (39%) had 25(OH)D levels below 12 ng/mL. Twenty-nine patients were enrolled in Graz and seven patients in Hoergas-Enzenbach. At baseline, data for body mass index, and urinary calcium to creatinine ratio were missing in one patient and we performed no data imputation for missing values. All other parameters presented in Table 1 were available in all study participants.

A total of 33 participants (mean (SD) age: 60 (9) years; 21% females; 25[OH]D: 15.6 (7.4) ng/mL) completed the baseline and follow-up visit. There was a significant effect of vitamin D supplementation on 25(OH)D with a mean treatment effect (95% CI) of 15.2 (8.0 to 22.4) ng/mL (*p* < 0.001). For all secondary outcomes, there was no significant treatment effect (Table 2).

Regarding parameters of mineral metabolism, we observed no significant effect on PTH levels (mean treatment effect (95% CI): −2.3 (−10.0 to 5.4) pg/mL; *p* = 0.548) and any other parameter of mineral metabolism (Table 3). No patient died during the study and there was no excess of adverse events in the vitamin D group.

## 4. Discussion

In this RCT in patients with compensated cirrhosis, we have shown that vitamin D supplementation significantly increases 25(OH)D serum concentrations but there was no significant effect neither on parameters of liver function and fibrosis nor on parameters of mineral metabolism including PTH.

Our study is, to the best of our knowledge, the first vitamin D RCT in cirrhotic patients. Considering the central role of the liver in vitamin D metabolism, it was still an important research question to evaluate whether and to what extent vitamin D supplementation increases serum 25(OH)D concentrations in the setting of cirrhosis. Importantly, the treatment effect (with 95% CI) on 25(OH)D levels was 15.2 (8.0 to 22.4) ng/mL, and thus similar to the treatment effect of 11.5 (9.4 to 13.7) ng/mL that we observed in a RCT of hypertensive patients using exactly the same vitamin D intervention in terms of dose and duration [34]. We can thus conclude from our results that in cirrhotic patients, the vitamin D doses required to achieve certain target levels of 25(OH)D are similar to those for other patient populations as well as for the general population. We observed, however, no significant effect of vitamin D supplementation on any liver function test, including parameters of liver synthetic function and hyaluronic acid as a marker of liver fibrosis. These findings should be interpreted with caution because the relatively small sample size and short duration of our RCT clearly limits the conclusions of these results. Nevertheless, we have to acknowledge that various clinical and experimental studies suggest that vitamin D may protect against liver damage by, e.g., metabolic, anti-inflammatory, and anti-fibrotic actions that warrant further studies addressing these issues [1,2,35]. Apart from this, the high prevalence of vitamin D deficiency in our study population is in line with previous reports and may be due to cirrhosis-associated factors such as low sunlight exposure, poor nutrition, low levels of vitamin D binding protein (DBP), increased 25(OH)D catabolism, and interruption of the enterohepatic circulation with reduced intestinal vitamin D absorption which is a bile-acid dependent process [18].

Considering that disturbances in calcium metabolism with a subsequent increase in PTH levels are a hallmark of vitamin D deficiency, we further evaluated whether vitamin D supplementation exerts any effect on these parameters. Interestingly, in contrast to established knowledge in other populations and our previous RCT in hypertensive patients, there was no vitamin D effect on any parameter of mineral metabolism in our cohort of patients with cirrhosis [34]. In particular, the missing effect on PTH is of interest because a decrease in PTH levels after vitamin D supplementation indicates beneficial effects of vitamin D on mineral metabolism. In this context, it should also be stressed that the classic definition of vitamin D deficiency is based on the fact that PTH increases below certain 25(OH)D levels and that the basis for vitamin D intake recommendations for the general population are the effects of vitamin D on mineral metabolism and bone health with a protection against osteomalacia [3,4]. Our findings, along with the results from some previous uncontrolled vitamin D intervention studies, question whether vitamin D supplementation in cirrhosis patients has a clinically relevant effect on PTH homeostasis [2]. Underlying mechanisms for the missing effect on PTH remain speculative, but it has been hypothesized that in patients with cirrhosis, total 25(OH)D concentrations may not be the most accurate marker for physiologically-active vitamin D [36]. In view of existing literature suggesting that patients with liver diseases suffer from a relatively high fracture incidence and a low bone mineral density along with a disturbed bone turnover, it could be hypothesized that a PTH-independent process, such as increased calcium mobilization from the skeleton, ensures physiological plasma calcium levels that prevent an increase in PTH, even under conditions of vitamin D deficiency [1,2,37]. Nevertheless, it remains a challenge for the future to improve our understanding of the regulation of bone and mineral metabolism in patients with cirrhosis and to evaluate whether vitamin D supplementation in these patients exerts some beneficial effects on skeletal health. Our findings may aid in the design of future RCTs in this field because our study results suggest that the increase in 25(OH)D in cirrhotic patients is similar when compared to other patient cohorts or the general population. In this context, it should also be noted that although we did not observe beneficial effects of vitamin D supplementation on several outcome measures, there were, on the other hand, also no adverse effects of our intervention. This underlines the safety of vitamin D supplementation in patients with cirrhosis. This is of clinical relevance because several guidelines for patients with liver diseases actually recommend vitamin D supplementation despite the lack of firm evidence base [2].

Our study is limited by a relatively small sample size, and the null findings of our secondary outcomes must thus be interpreted with caution as they are prone to statistical type 2 errors; that is, failing to detect an effect that is actually present. Our results are derived from a specific population of patients with compensated cirrhosis of mainly alcoholic origin in Austria and may not be necessarily generalizable to other cirrhotic populations. We used a certain vitamin D intervention and we cannot rule out that other vitamin D treatment modalities in terms of formulation, dosage, or treatment period produce different results. In addition, we measured 25(OH)D by means of an immunoassay and not by the reference method; *i.e.*, liquid chromatography-mass spectrometry (LC-MS) with the opportunity to detect specific 25(OH)D metabolites such as the C3-epimer [38]. We also did not measure some additional parameters of vitamin D and calcium metabolism, such as DBP or fibroblast growth factor 23 (FGF-23) [39]. Furthermore, our study is also limited because we did not address clinical outcomes. Main strengths of our work are that this is the first vitamin D RCT in patients with cirrhosis and that our study is an officially registered trial (ClinicalTrials.gov Identifier: NCT02009748) with pre-specified outcome measures.

In conclusion, we could show in this RCT that vitamin D supplementation increases 25(OH)D serum concentrations in patients with cirrhosis. There was, however, no significant vitamin D effect on parameters of mineral metabolism, liver function, or fibrosis. It remains a challenge for the future to evaluate in larger and longer studies whether vitamin D supplementation in cirrhotic patients exerts beneficial effects on bone and mineral metabolism or other relevant outcomes in order to provide a sound scientific basis for vitamin D recommendations in the setting of chronic liver failure.

## Figures and Tables

**Table 1:** Baseline characteristics of all randomized study participants.

Characteristics	All (*n* = 36)	Vitamin D (*n* = 18)	Placebo (*n* = 18)	*p* Value
Females (%)	25	33	17	0.248
Age (years)	61 ± 9	63 ± 9	58 ± 9	0.110
Body mass index (kg/m²)	26.9 ± 4.4	27.9 ± 5.2	26.0 ± 3.3	0.204
Alcoholic cirrhosis (%)	64	67	61	0.729
MELD score	9 (8–12)	10 (8–12)	9 (7–10)	0.136
Child Pugh score	5 (5-6)	5 (5–6)	5 (5–5)	0.570
AST (IU/L)	39 (30–61)	52 (32–61)	37 (26–59)	0.448
ALT (IU/L)	34 (22–46)	35 (27–44)	29 (20–49)	0.874
GGT (IU/L)	106 (71–201)	97 (46–240)	113 (74–180)	0.967
AP (IU/L)	99 (79–127)	105 (78–149)	93 (80–114)	0.341
Albumin (g/dL)	4.0 ± 0.5	4.0 ± 0.4	4.0 ± 0.6	0.642
INR	1.21 (1.09–1.35)	1.29 (1.15–1.36)	1.17 (1.08–1.32)	0.102
Bilirubin (mg/dL)	0.8 (0.6–1.3)	1.0 (0.7–1.4)	0.7 (0.5–1.1)	0.104
Hyaluronic acid (ng/mL)	270 (83–536)	328 (166–1000)	266 (67–488)	0.164
ELF score	11.2 ± 1.3	11.5 ± 1.3	10.9 ± 1.1	0.125
Creatinine (mg/dL)	0.85 (0.78–0.99)	0.85 (0.76–0.99)	0.86 (0.77–1.01)	0.864
eGFR (ml/min/1.73 m²)	92 ± 25	89 ± 23	94 ± 27	0.521
Platelet count (10^9^/L)	116 (87–153)	116 (70–155)	121 (90–162)	0.661
Triglycerides (mg/dL)	118 (76–149)	105 (69–150)	121 (89–146)	0.944
HDL-cholesterol (mg/dL)	44 ± 15	42 ± 17	45 ± 13	0.557
LDL-cholesterol (mg/dL)	100 ± 32	95 ± 31	105 ± 34	0.396
Total cholesterol (mg/dL)	172 ± 41	166 ± 43	178 ± 40	0.420
CRP (mg/L)	3.2 (1.2–7.4)	2.7 (1.2–7.9)	3.7 (1.1–6.8)	0.988
25(OH)D (ng/mL)	15.7 ± 7.2	15.9 ± 7.5	15.5 ± 7.0	0.882
PTH (pg/mL)	40.7 ± 15.5	39.2 ± 11.2	42.1 ± 19.0	0.596
Total plasma calcium (mmol/L)	2.33 ± 0.11	2.32 ± 0.09	2.35 ± 0.12	0.557
Free plasma calcium (mmol/L)	1.16 ± 0.05	1.16 ± 0.05	1.16 ± 0.04	1.000
Plasma phosphate (mmol/L)	1.01 ± 0.19	1.00 ± 0.22	1.02 ± 0.16	0.834
Urinary calcium/creatinine ratio	0.26 (0.16–0.36)	0.31 (0.14–0.46)	0.25 (0.16–0.30)	0.608
Plasma sodium (mmol/L)	139 (137–141)	139 (138–140)	139 (137–141)	0.869
Calcium/Vitamin D supplement (%)	6	11	0	0.486
Diuretics (%)	53	61	44	0.317

Date are presented as means with standard deviation, medians with interquartile range or as percentages. Comparisons between the vitamin D and placebo group were calculated with Student´s *t*-test or with Chi Square test and Fisher’s exact test. MELD: model for end-stage liver disease; AST: Aminotransferase; ALT: Alanine aminotransferase; GGT: Gamma glutamyltransferase; AP: Alkaline phosphatase; INR: International Normalized Ratio; ELF: Enhanced liver fibrosis; eGFR: estimated glomerular filtration rate; HDL: High-density lipoprotein; LDL: Low-density lipoprotein; 25(OH)D: 25-hydroxyvitamin D; PTH: Parathyroid hormone.

**Table 2:** Outcome variables at baseline and follow-up and changes from baseline in study participants with available values at both study visits.

Characteristics	Baseline	Follow-up	Mean Change from Baseline	Treatment Effect	*p* Value
25-hydroxyvitamin D (ng/mL)					
Vitamin (*n* = 16)	15.8 ± 7.8	33.8 ± 10.3	18.0 (11.4 to 24.6)	15. 2 (8.0 to 22.4)	<0.001
Placebo (*n* = 16)	15.8 ± 7.4	18.6 ± 9.9	2.8 (−2.3 to 7.9)		
AST (IU/L) #					
Vitamin (*n* = 16)	52 (33–62)	51 (35–67)	1 (−7 to 10)	0 (−13 to 13)	0.663
Placebo (*n* = 17)	37 (25–61)	47 (28–61)	2 (−12 to 16)		
ALT (IU/L) #					
Vitamin (*n* = 16)	39 (29–46)	36 (29–52)	0 (−5 to 6)	−1 (−17 to 14)	0.962
Placebo (*n* = 17)	28 (20–46)	30 (20–39)	1 (−13 to 16)		
GGT (IU/L) #					
Vitamin (*n* = 16)	97 (44–314)	100 (55–288)	4 (−17 to 24)	1 (−36 to 38)	0.440
Placebo (*n* = 17)	122 (74–192)	139 (56–225)	4 (−27 to 35)		
AP (IU/L) #					
Vitamin (n=16)	112 (79–176)	124 (75–180)	5 (−8 to 18)	8 (−10 to 25)	0.700
Placebo (n=17)	88 (80–118)	98 (75–105)	−2 (−13 to 8)		
Albumin (g/dL)					
Vitamin (*n* = 16)	4.0 ± 0.4	3.9 ± 0.5	−0.2 (−0.4 to 0.0)	−0.1 (−0.3 to 0.1)	0.366
Placebo (*n* = 17)	4.0 ± 0.6	4.0 ± 0.5	−0.1 (−0.2 to 0.2)		
INR #					
Vitamin (*n* = 16)	1.27 (1.14–1.39)	1.24 (1.12–1.45)	0.08 (−0.11 to 0.26)	0.06 (−0.14 to 0.25)	0.610
Placebo (*n* = 15)	1.18 (1.08–1.39)	1.18 (1.10–1.29)	0.00 (−0.05 to 0.04)		
Bilirubin (mg/dL) #					
Vitamin (*n* = 16)	1.1 (0.8–1.5)	1.0 (0.8–1.4)	−0.2 (−0.5 to 0.2)	0.2 (−0.1 to 0.4)	0.628
Placebo (*n* = 17)	0.7 (0.5–1.1)	0.6 (0.5–1.0)	−0.2 (−0.4 to 0.1)		
Hyaluronic acid (ng/mL) #					
Vitamin (*n* = 16)	328 (182–911)	367 (135–844)	30 (−44 to 104)	−39 (−155 to 77)	0.599
Placebo (*n* = 15)	266 (72–517)	411 (78–593)	75 (−16 to 166)		
ELF score					
Vitamin (*n* = 16)	11.5 ± 1.2	11.7 ± 1.2	0.2 (−0.1 to 0.4)	0.0 (−0.4 to 0.3)	0.836
Placebo (*n* = 16)	10.9 ± 1.1	11.1 ± 1.2	0.2 (0.0 to 0.5)		

Data at baseline and follow-up are shown as means with standard deviation or as medians with interquartile range. Change from baseline data are shown as means (with 95% confidence interval); Treatment effects (with 95% confidence intervals) and *p* values were calculated by Analysis of Co-Variance (ANCOVA) for group differences at follow-up adjusted for baseline values; # skewed variable for which logarithmic transformed values were used in ANCOVA but untransformed values are shown in the Table.

**Table 3:** Parameters of mineral metabolism at baseline and follow-up, and changes from baseline in study participants with available values at both study visits.

Characteristics	Baseline	Follow-up	Mean Change from Baseline	Treatment Effect	*p* Value
PTH (pg/mL)					
Vitamin (*n* = 16)	38.8 ± 11.3	38.5 ± 15.1	−0.3 (−8.0 to 7.3)	−2.3 (−10.0 to 5.4)	0.548
Placebo (*n* = 16)	41.5 ± 20.1	42.6 ± 15.3	1.1 (−3.3 to 5.5)		
Total plasma calcium (mmol/L)					
Vitamin (*n* = 16)	2.33 ± 0.09	2.27 ± 0.07	−0.06 (−1.0 to −0.02)	0.00 (−0.05 to 0.04)	0.849
Placebo (*n* = 17)	2.34 ± 0.11	2.28 ± 0.12	−0.05 (-0.09 to 0.02)		
Free plasma calcium (mmol/L)					
Vitamin (*n* = 16)	1.15 ± 0.05	1.15 ± 0.05	−0.01 (−0.03 to 0.02)	0.02 (−0.02 to 0.05)	0.254
Placebo (*n* = 17)	1.15 ± 0.05	1.13 ± 0.06	−0.02 (−0.05 to 0.00)		
Urinary midstream calcium to creatinine ratio #					
Vitamin (*n* = 13)	0.31 (0.12–0.43)	0.29 (0.14–0.54)	−0.06 (−0.31 to 0.18)	0.00 (−0.27 to 0.28)	0.354
Placebo (*n* = 17)	0.25 (0.19–0.31)	0.19 (0.09–0.29)	0.04 (−0.18 to 0.25)		
Plasma phosphate (mmol/L)					
Vitamin (*n* = 16)	0.99 ± 0.22	1.03 ± 0.20	0.04 (−0.05 to 0.12)	0.04 (−0.60 to 0.13)	0.448
Placebo (*n* = 17)	1.02 ± 0.17	1.01 ± 0.14	−0.01 (−0.09 to 0.07)		

Data at baseline and follow-up are shown as means with standard deviation or as medians with interquartile range. Change from baseline data are shown as means (with 95% confidence interval); Treatment effects (with 95% confidence intervals) and *p* values were calculated by Analysis of Co-Variance (ANCOVA) for group differences at follow-up adjusted for baseline values; # skewed variable for which logarithmic transformed values were used in ANCOVA but untransformed values are shown in the Table.
